# Reflective learning conversations model for simulation debriefing: a co-design process and development innovation

**DOI:** 10.1186/s12909-023-04778-0

**Published:** 2023-11-07

**Authors:** Emad Almomani, Jacqueline Sullivan, Omar Saadeh, Emad Mustafa, Natalie Pattison, Guillaume Alinier

**Affiliations:** 1 Hamad Medical Corporation, HMC-Qatar, PO Box: 3050, Doha, Qatar; 2 University of Hertfordshire-School of Health and Social Work, Hatfield, Hertfordshire AL10 9AB UK; 3 Weill Cornell Medicine-Qatar, Doha, Qatar; 4 Northumbria University, Newcastle, UK

**Keywords:** SBE, Clinical reasoning, Reflective learning conversations model, Post-simulation debriefing

## Abstract

**Background:**

Health practitioners must be equipped with effective clinical reasoning skills to make appropriate, safe clinical decisions and avoid practice errors. Under-developed clinical reasoning skills have the potential to threaten patient safety and delay care or treatment, particularly in critical and acute care settings. Simulation-based education which incorporates post-simulation reflective learning conversations as a debriefing method is used to develop clinical reasoning skills while patient safety is maintained. However, due to the multidimensional nature of clinical reasoning, the potential risk of cognitive overload, and the varying use of analytic (hypothetical-deductive) and non-analytic (intuitive) clinical reasoning processes amongst senior and junior simulation participants, it is important to consider experience, competence, flow and amount of information, and case complexity related factors to optimize clinical reasoning while attending group- based post-simulation reflective learning conversations as a debriefing method. We aim to describe the development of a post-simulation reflective learning conversations model in which a number of contributing factors to achieve clinical reasoning optimization were addressed.

**Methods:**

A Co-design working group (N = 18) of doctors, nurses, researchers, educators, and patients’ representatives collaboratively worked through consecutive workshops to co-design a post-simulation reflective learning conversations model to be used for simulation debriefing. The co-design working group established the model through a theoretical and conceptual-driven process and multiphasic expert reviews. Concurrent integration of appreciative inquiry, plus/delta, and Bloom’s Taxonomy methods were considered to optimize simulation participants’ clinical reasoning while attending simulation activities. The face and content validity of the model were established using the Content Validity Index CVI and Content Validity Ratio CVR methods.

**Results:**

A Post-simulation reflective learning conversations model was developed and piloted. The model was supported with worked examples and scripted guidance. The face and content validity of the model were evaluated and confirmed.

**Conclusions:**

The newly co-designed model was established in consideration to different simulation participants’ seniority and competence, flow and amount of information, and simulation case complexity. These factors were considered to optimize clinical reasoning while attending group-based simulation activities.

## Background

Clinical reasoning is considered as a backbone of health care clinical practice [1, 2], and an essential element of clinical competence [1, 3, 4]. It is a reflective process that healthcare practitioners use to identify and perform the most appropriate intervention for each clinical situation they encounter [5, 6]. Clinical reasoning is described as a complex cognitive process that uses formal and informal thinking strategies to gather and analyze patient information, evaluate the significance of this information, and determine the value of alternative actions [7, 8]. It depends upon the ability to collect cues, process information, and understand patient problems to take the right action, for the right patient, at the right time, for the right reason [9, 10].

All healthcare providers are faced with making complex decisions in situations where there is a high degree of uncertainty [11]. In critical and acute care practice, clinical situations and emergencies arise where immediate reactions and interventions are essential to save lives and to maintain patient safety [12]. Under-developed clinical reasoning skills and a lack of competence in critical and acute care practices are associated with higher rates of clinical errors, delay of care or treatment [13], and patient safety risks [14–16]. To avoid practice errors, healthcare practitioners must be competent and well-equipped with effective clinical reasoning skills for safe and appropriate decision-making [16–18]. The non-analytic (intuitive) reasoning process is a fast-track process, which is preferred by expert health practitioners. In comparison, the analytic (hypothetical-deductive) reasoning process, which is slower and more deliberate in nature, is more commonly used by less experienced practitioners [2, 19, 20]. Taking into consideration the complexity of healthcare clinical environments and the potential risk for practice errors [14–16], Simulation-Based education (SBE) is commonly used to give a chance for healthcare practitioners to develop competence and clinical reasoning skills in a safe environment, and to be exposed to various case complexities while patient safety is maintained [21–24].

Simulation is defined by the Society for Simulation in Healthcare (SSH) as a “technique that creates a situation or environment to allow persons to experience a representation of a real event for the purpose of practice, learning, evaluation, testing, or to gain understanding of systems or human actions” [23]. Well-structured simulation activities give participants a chance to be immersed in scenarios that mimic clinical situations, simultaneously mitigating safety risks [24, 25], and to practice clinical reasoning through focused learning opportunities [21, 24, 26–28]. SBE augments on-site clinical experiences by exposing learners to clinical experiences they may not have experienced in a real-life patient environment [24, 29]. It is a non-threatening, blame-free, controlled, low-risk, and safe learning environment that encourages the development of knowledge, clinical skills, competence, critical thinking, and clinical reasoning [22, 29–31], and it helps healthcare professionals overcome the emotional strain of a situation to enhance learning [22, 27, 28, 30, 32].

To support the effective development of clinical reasoning and decision-making skills through SBE, attention must be given to the design, modalities, and structure of post-simulation debriefing processes [24, 33–35]. Post-simulation reflective learning conversations (RLC) are used as a debriefing method to help participants to reflect, explain actions, and in the context of teamwork to use the peer support and power of group-think [32, 33, 36]. Using group-based RLC is associated with a potential risk of underdeveloped clinical reasoning especially with different participants’ competence and seniority levels. The dual-process framework described the multidimensional nature of clinical reasoning, and the variation in the tendency to use analytic (hypothetical-deductive) reasoning processes by senior health practitioners and non-analytic (intuitive) reasoning processes by junior health practitioners [34, 37]. These dual reasoning processes are associated with a challenge of the best reasoning process to fit different situations, and it is unclear and debatable how analytical and non-analytical can be effectively used in the presence of senior and junior participants within the same simulation group, and even for groups of seniors and juniors but with different competence and experience levels attending different simulation scenario complexities [34, 37]. That multidimensional nature of clinical reasoning is associated with a potential risk of underdeveloped clinical reasoning and cognitive overload especially when practitioners attend group-based SBEs with different case complexity and seniority levels [38]. Importantly, despite the availability of many simulation debriefing models using RLC, none of these were developed with a specific focus on developing clinical reasoning skills in consideration of experience, competence, flow and amount of information, and simulation case complexity factors [38, 39]. All of that brought the need to develop a structured model, which takes account of different contributing and influencing factors to optimize clinical reasoning while attending post-simulation RLC as a debriefing method. We describe a co-design and development process of theoretically and conceptually driven post-simulation RLC. A model was developed to optimize clinical reasoning skills while attending SBE taking into consideration a wide range of contributing and influencing factors to achieve clinical reasoning development optimization.

## Methods

A post-simulation RLC model was co-designed drawing on existing models and theories of clinical reasoning, reflective learning, education, and simulation. A collaborative working group (N = 18) was established to co-design the model, which consisted of 10 critical care nurses from a range of grades, experience, and gender, one critical care physician, three patient representatives who had previously been admitted to a critical care unit, 2 researchers, and 2 senior nurse educators. This co-design innovation was devised and developed as a result of an equal partnership of stakeholders who have a lived experience of healthcare, either as healthcare professionals who were involved in the development of the proposed model, or other stakeholders, such as patients [40–42]. Including patient representatives in the co-design process adds further value to the process as the ultimate aim of the initiative is to enhance patient care and safety [43].

**Fig. 1:**
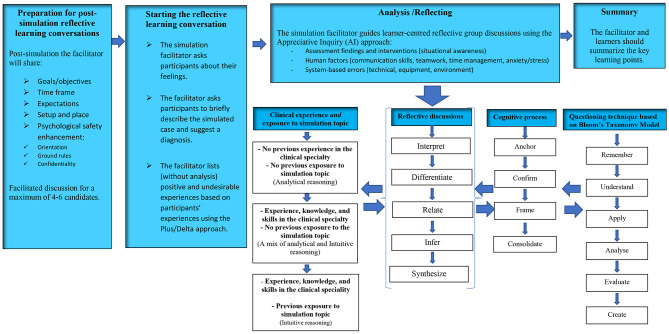
Post-simulation Reflective Learning Conversations RLC Model

The working group conducted six 2–4-hour workshops which focussed on developing the model structure, flow, and content. The workshops included discussions, exercises, and activities to establish the model. Elements of the model were underpinned by a range of evidence-based resources, models, theories, and frameworks. Those included: constructivist learning theory [44]; dual loop framework [37]; clinical reasoning cycle [10]; Appreciative Inquiry (AI) method [45]; and the Plus/Delta debriefing method [46]. The model was co-designed in alignment with the International Nursing Association of Clinical and Simulation Learning INACSL standards of the debriefing process [36] and established to be self-explanatory incorporating worked examples. The model was developed and categorized into four phases: preparation for post-simulation reflective learning conversations; starting the reflective learning conversations; analysis /reflecting; and summary (Fig. 1). Details of each phase are discussed below.

The *preparation phase* of the model was established to mentally prepare the participants for the next phases, and to enhance participants’ active participation and engagement with secured psychological safety [36, 47]. This phase includes introducing the goals and objectives; expected duration of the RLC; expectations from both facilitators and participants during the RLC; orientation to venue and simulation setup; and enhancing and reinforcing psychological safety by exploring any concerning and distracting issues to participants and assuring confidentiality in a blame-free learning environment. The following representative responses by the co-design working group were considered to develop the RLC model preparation phase. Participant 7: “As a practicing junior nurse, if I attend a simulation activity with no previous background about the scenario in presence of seniors, I may avoid participating in the post-simulation conversations unless I feel that my psychological safety is secured, and I am protected without consequences”. Participant 4: “I believe having orientation and setting up the ground rules at early stages would help the simulation learners to be actively engaged during the post-simulation reflective learning conversations”.

The *starting phase of the RLC* model included exploring participants’ feelings; describing scenario’s main course and diagnosis; listing participants’ positive and undesirable experiences but without analysis. This phase of the model is established to trigger candidates to be self- and task-oriented, and mentally prepared for advanced analyses and in-depth reflection [24, 36]. It aimed to reduce the potential risk of cognitive overload [48] especially for those who are new to the simulation topic and without previous clinical experience of the skill/topic [49]. Asking participants to briefly describe the simulated case and suggest a diagnosis will help facilitator to ensure that group learners have the basic and generic understanding about the case before proceeding to advanced analysis/reflecting phase. Moreover, asking participants at this phase to share their feelings during simulation scenarios would help them to overcome emotional strain of a situation to enhance learning [24, 36]. Addressing emotions will also help the RLC facilitators to understand how participant’s’ feelings affected the individual and group performances, and these can be critically discussed during the reflecting/analysis phase. The Plus/Delta method was embedded into this phase of the model as a preparatory and critically important step for reflecting/analysis phase [46]. Through using the Plus/Delta method both participants and learners can address/list their observations, feelings, and simulation experiences which then can be discussed point by point during reflecting/ analysis phase of the model [46]. That would help participants to achieve metacognition status with focused and prioritized learning opportunities toward clinical reasoning optimization [24, 48, 49]. The following representative responses by the co-design working group were considered to develop the RLC model starting phase. Participant 2:” I believe as a patient who was previously admitted in the critical care units that we should address the feeling and emotions of simulation learners, I am raising this up because during my admission period, I observed a high level of stress and anxiety among healthcare practitioners, especially, during the critical and emergency situations. The stress and emotions during the simulation experience need to be considered in this model”. Participant 16: “For me as an educator, I think it is very important to incorporate the Plus/Delta method so learners will be encouraged to be actively engaged by mentioning good things they faced during the simulation scenario and areas for improvement”.

Despite the critical importance of previous phases of the model, the *analysis/reflecting phase* is the most important to achieve clinical reasoning optimization. It was established to achieve advanced analysis/synthesis and deep reflection in consideration to clinical experience, competence, and exposure to simulation topics; flow and structure of RLC; amount of delivered information to avoid cognitive overload; effective use of reflective questioning technique to achieve learner-centeredness and active learning. In this phase, the clinical experience and exposure to simulation topic was categorized into three sections to match different experience and competence levels; first: no previous experience in the clinical specialty/ no previous exposure to simulation topic, second: experience, knowledge, and skills in the clinical specialty/ no previous exposure to the simulation topic, and third: experience, knowledge, and skills in the clinical speciality/ previous exposure to simulation topic. This was classified to meet demands of different experiences and competence levels within the same group, therefore balancing the tendency of less experienced practitioners to use analytical reasoning in comparison to more experienced ones who tend to use non-analytical reasoning skills [19, 20, 34, 37]. The flow of the RLC was developed in a structured way based on the clinical reasoning cycle [10], reflective simulation framework [47], and experiential learning theory [50]. That was achieved through a sequential process of; interpret, differentiate, relate, infer, and synthesize.

To avoid cognitive overload, facilitate learner-centred and reflective conversation process with adequate time, and give chances to participants to reflect, analyse, and synthesise to achieve confidence were considered. The cognitive process during the RLC was addressed based on the dual loop framework [37] and cognitive load theory [48] through a process of anchoring, confirming, framing, and consolidating. Having a structured flow of conversations and giving adequate time to reflect considering both experienced and non-experienced participants would reduce the potential risk of cognitive load especially after complex simulation scenarios with different participants’ previous experience, exposure, and competence levels. The reflective questioning technique of the model was established based on Bloom’s taxonomy model [51] and Appreciative Inquiry (AI) [45] method in which the simulation facilitators question in incremental, Socratic, and reflective way starting with knowledge related questions toward skills and reasoning related questions. This questioning technique would encourage participants to be actively engaged and to incrementally reflect with low risk of cognitive overload, therefore enhancing clinical reasoning optimization. The following representative responses by the co-design working group were considered to develop the RLC model analysis/reflecting phase. Participant 13: “To avoid cognitive overload, we need to consider the amount and flow of information while attending the post-simulation learning conversations, for that, I think giving enough time for learners to reflect is crucial, and starting the conversation with basic knowledge and skills and then incrementally discussing the higher levels of knowledge and skills to achieve metacognition”. Participant 9: “I do strongly believe that questioning technique using Appreciative Inquiry (AI) method and reflective questions using Bloom’s Taxonomy model would encourage active learning and learner-centredness, at the same time, will reduce the potential risk of cognitive overload”. The *summary phase* of the model aimed to summarize the key learning points raised during the RLC and to ensure that learning objectives are achieved. Participant 8: “It is very important that both learners and facilitators to agree on the most important take home messages, and the critical aspects that should be considered to achieve transference into practice”.

Ethical approvals were obtained with protocol numbers (MRC-01-22-117) and (HSK/PGR/UH/04728). The model was piloted in three critical care simulation-based specialty courses to evaluate model usability and practicality. The model face validity was evaluated by the co-design working group (N = 18), and by educational experts working as directors of education (N = 6) to amend appearance, grammatical and flow related issues. Following face validity, content validity was evaluated by senior nurse educators (N = 6) certified by the American Nurse Credentialing Center (ANCC) and working as educational planners, and (N = 6) directors of education with more than 10 years of educational and simulation experiences. The content validity was conducted using a Content Validity Ratio (CVR) and a Content Validity Index (CVI). The CVI was assessed using Lawshe’s method [52] and CVR assessed using Waltz and Bausell ‘s method [53]. The CVR items were essential, useful but not essential, and not essential. The CVI was scored based on a four-point scale to address relevancy, simplicity, and clarity where 1 = irrelevant, 2 = relatively relevant, 3 = relevant and 4 = highly relevant. After ensuring face and content validities, awareness and orientational sessions in addition to hands on workshops were conducted to the educators who are going to use the model.

## Results

**Fig. 2:**
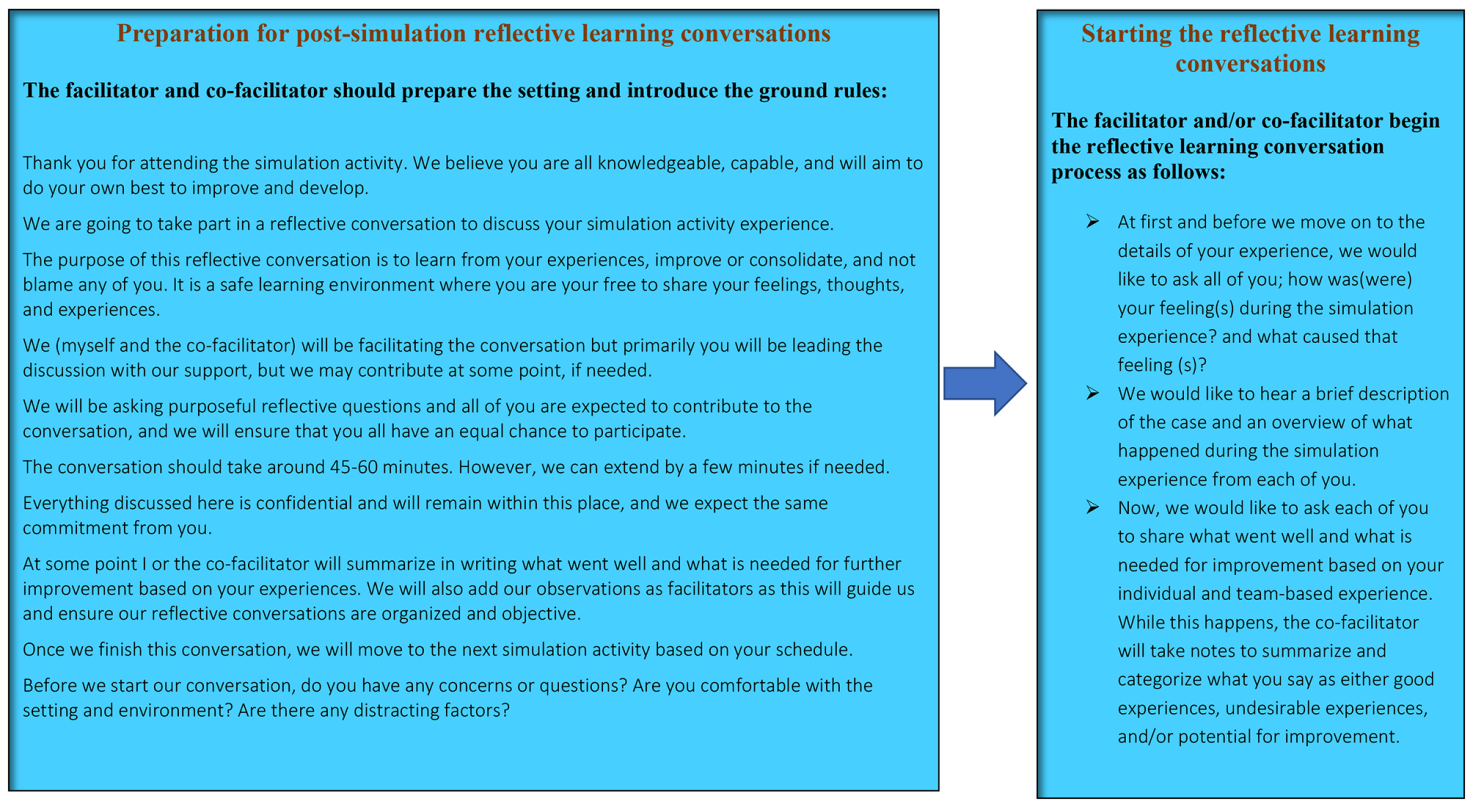
Post-simulation Reflective Learning Conversations RLC Model Script/Example

**Fig. 3:**
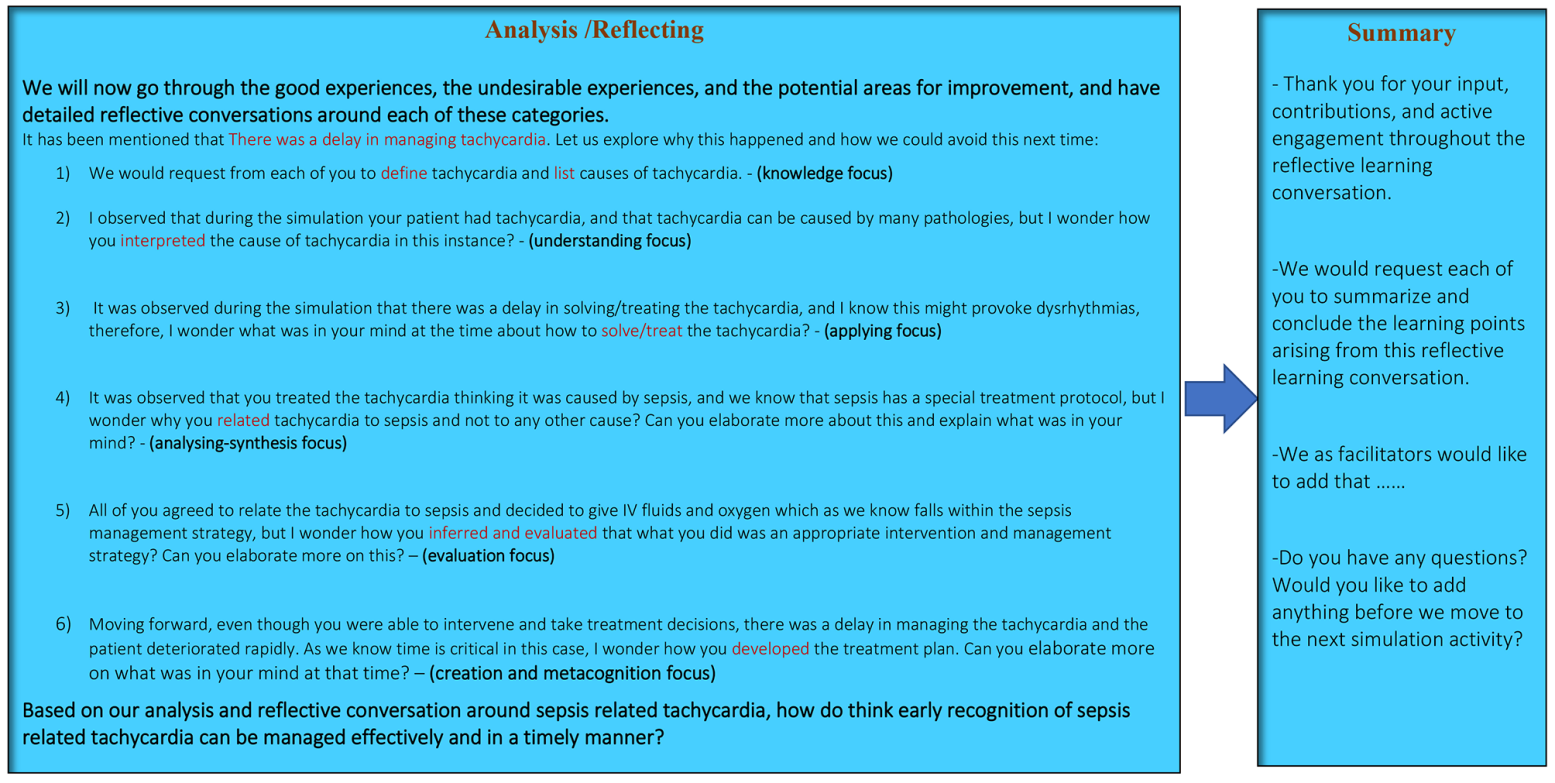
Post-simulation Reflective Learning Conversations RLC Model Script/Example, cont.

The working group was able to produce and pilot a post-simulation RLC model to optimize the clinical reasoning skills while attending critical care SBEs (Figs. 1, 2 and 3). The CVR = 1.00, and the CVI = 1.00, reflecting appropriate face and content validities [52, 53].

## Discussions

The model was established to fit group- based SBE in which immersive and complex scenarios are used for participants with same or different experience, exposure, and seniority levels. The RLC conceptual model was developed in alignment with the INACSL simulation standards of simulation debriefing [36] and designed to be learner-centered and self-explanatory incorporating worked examples (Figs. 1, 2 and 3). The model was purposefully developed and categorized into four phases to meet the simulation standards by starting with briefing followed by reflective analysis/synthesis and ended with take home messages and summary. To avoid potential risks of cognitive overload, each phase of the model was purposefully developed as a prerequisite to next phase [34].

The impact of seniority and group harmony factors while attending RLC have not previously investigated [38]. Taking in account the practical concepts of dual loop and cognitive overload theories into simulation practices [34, 37], it is important to consider that attending group- based SBEs with different participants’ experience and competence levels within same simulation learning group is a challenge. Ignoring the amount of information, flow and structure of teaching delivery, and concurrent use of fast and slow cognitive reasoning processes by seniors and juniors, are associated potential risk of cognitive overload [18, 38, 46]. These factors were considered in the development of the RLC model to avoid underdeveloped and/or suboptimal clinical reasoning [18, 38]. For that, it is important to take in account that conducting RLC with different seniority and competence levels provokes domination effect by senior participants. That could happen due to tendency of senior participants to escape the basic concepts of learning which may be critical to junior participants to achieve metacognition and to move to higher level of thinking and reasoning process [38, 47]. The RLC model was designed to engage both seniors and junior nurses through the appreciative inquiry and plus delta methods [45, 46, 51]. By using these methods, the inputs of both senior and junior participants with different competence and experience levels will be all listed and reflectively discussed point by point by the debriefing facilitator and co-facilitator [45, 51]. The debriefing facilitators would add their inputs in addition to simulation participants’ inputs, consequently, all collective observations would comprehensively cover each learning point, therefore, metacognition enhancement toward clinical reasoning optimization [10].

The flow of information and structure of teaching delivery using the RLC model were considered through a systematic and multiphasic processes. That design aimed to help debriefing facilitators ensuring that each participant is clear and confident at each phase before moving to next phases. The facilitator will be able to trigger reflective discussions to engage all participants, and to reach a point that participants with different seniority and competence level are agreed to best practice of each discussion point before moving to next [38]. Using that way would help experienced and competent participants to share their inputs/observations whereas the inputs/observations of less experienced and competent participants are appreciated and discussed [38]. However, achieving that would challenge the facilitator on how to balance the discussions and to give equal chances for senior and junior participants. For that, the questioning technique of the model was purposefully developed using Bloom’s taxonomy model incorporating the Appreciative Inquiry and Plus/ Delta methods [45, 46, 51]. Using these methods and starting with knowledge and understanding focus questions/ reflective discussions would encourage less experienced participants to participate and be actively engaged in the discussions, which after, the facilitator will gradually move to higher level of evaluation and synthesis questions/discussions in which both senior and junior participants should be given equal chances to participate based on their previous exposure and experiences to either clinical skill or simulation scenario. This way would help less experienced participants to be actively engaged and to benefit from shared experience by more experienced participants and the input of debriefing facilitators. On other hand, the model was not designed only to fit the SBEs with different participants competence and experience levels, but also when SBE group participants have same experience and competence levels. The model developed to enhance smooth and systematic movement transition of the group from knowledge and understanding focus to synthesis and evaluation focus to achieve the learning objectives. The model structure and flow were designed to fit simulation groups with different and same competence and experiences levels.

Moreover, despite that healthcare SBE incorporating RLC is used to develop clinical reasoning and competence for healthcare practitioners [22, 30, 38], however, the associated factors in relation to case complexity and potential risk of cognitive overload need to be considered, especially when participants attend SBE scenarios mimic critically ill patients with high complexity that need immediate interventions and critical decisions [2, 18, 37, 38, 47, 48]. For that, it is critical to consider while attending SBEs the tendency of experienced and less experienced participants’ to concurrently shift between analytical and non-analytical reasoning systems, and to establish an evidence-based methods that keep both seniors and juniors actively engaged in the learning process. Therefore, the model was developed that whatever simulation case complexity is introduced, the facilitator should ensure that knowledge and basic understanding aspects are covered at first for both senior and junior participants, and then to progress incrementally and reflectively to facilitate the analysis, synthesis, and evaluation aspects. This will help junior to build up and consolidate learning, and at the same time seniors to synthesize and develop new learning. That would meet reasoning process demands of each participant with respect to previous experiences and competence, and to have a universal format that fit the tendency of seniors and juniors to concurrently shift between analytical and non- analytical reasoning systems, consequently, clinical reasoning optimization.

Moreover, simulation facilitators/debriefers may struggle to master simulation debriefing skills. Using a cognitive debriefing script is deemed effective to increasing facilitators’ knowledge acquisition and behavioural skills compared with those facilitators who did not use a script [54]. Script is a cognitive aid that may promote simulation faculty development efforts and augment debriefing skills particularly in those educators who are still solidifying their debriefing expertise [55], therefore, scripted worked examples were added to the model to enhance simulation faculty development, and to achieve higher practicality and to develop a friendly user model. (Figures 2 and 3).

The concurrent integration of Plus/Delta, Appreciative Inquiry, and Bloom’s Taxonomy questioning methods were not previously addressed in currently available simulation debriefing and guided reflection models. The integration of these methods highlights the innovative aspects of the RLC model in which these methods were integrated in a universal format to achieve clinical reasoning optimization and learner-centredness. Medical educators can benefit from using the RLC model for simulation debriefing of group- based SBEs to enhance and optimize participants’ clinical reasoning development. The scripts of the model may help the educators to master the reflective debriefing process and to consolidate their skills in being confident and competent debriefing facilitators.

SBEs may incorporate a wide range of different modalities and methods including but not limited to mannequins based SBE, task trainers, patient simulators, standardized patients, virtual and augmented reality. Taking in account that debriefing is one of the essential simulation standards, the post- simulation RLC model can be used as a debriefing model while using these modalities. Moreover, despite that the model was developed for nursing discipline but also potential to be used for interprofessional healthcare SBEs, highlighting the need for future research initiatives to validate the RLC model for interprofessional education.

### Limitations

- The post- simulation RLC model was developed and evaluated to be used for critical care nursing SBEs. Future evaluations/ validations of the model to enhance the generalizability level of the model be used for other health care disciplines and interprofessional SBEs are recommended.
- The model was developed through theoretical and conceptual driven process by a co-design working group. To enhance the validity and generalizability levels of the model, advanced reliability measures using comparative studies can be considered in the future.

## Conclusions

To minimize practice errors, health care practitioners must be competent with effective clinical reasoning skills to ensure safe and appropriate clinical decision-making. SBE incorporating RLC as a debriefing method promotes the development of knowledge and practise-focused skills necessary to develop clinical reasoning. However, the multidimensional nature of clinical reasoning associated with previous experience and exposure, variations in competence, amount and flow of information, and simulation scenario complexity, highlighted the importance of developing a post- simulation RLC model whereby clinical reasoning skills are actively and effectively embedded. Ignoring these factors may lead to underdeveloped and suboptimal clinical reasoning. The RLC model was established in consideration to these contributing and influencing factors to optimize clinical reasoning while attending group-based simulation activities. To achieve that the model concurrently integrated the use of appreciative inquiry, plus/delta, and Bloom’s Taxonomy methods.

## Data Availability

The datasets used and/or analyzed during the current study are available from the corresponding author on reasonable request.

## List of abbreviations

CVR: Content Validity Ratio
CVI: Content Validity Index
SSH: Society for Simulation in Healthcare
SBE: Simulation based Education
INACSL: International Nursing Association of Clinical and Simulation Learning
